# Relationship Between Pneumonia and Dysphagia in Patients With Multiple System Atrophy

**DOI:** 10.3389/fneur.2022.904852

**Published:** 2022-07-04

**Authors:** Ayako Wada, Michiyuki Kawakami, Yuka Yamada, Kentaro Kaji, Nanako Hijikata, Fumio Liu, Tomoyoshi Otsuka, Tetsuya Tsuji

**Affiliations:** ^1^Department of Rehabilitation Medicine, National Hospital Organization Higashisaitama National Hospital, Saitama, Japan; ^2^Department of Rehabilitation Medicine, Keio University School of Medicine, Tokyo, Japan

**Keywords:** pneumonia, videofluorographic, reliability, validity, neurodegenerative disease

## Abstract

**Introduction:**

Dysphagia is one of the most clinically significant disabilities in patients with multiple system atrophy (MSA), because it can cause aspiration pneumonia, which is potentially fatal. In this study, the Neuromuscular disease Swallowing Status Scale (NdSSS), which was developed to evaluate dysphagia in patients with neuromuscular diseases, was used to evaluate patients with MSA. In addition, correlation between a history of pneumonia and swallowing function was evaluated.

**Methods:**

Study 1: Reliability, concurrent validity, and responsiveness of the NdSSS in patients with MSA. In 81 patients for whom evaluation items could be collected, the NdSSS was tested for its interrater and intrarater reliability using weighted kappa statistics. Concurrent validity was assessed by correlating the NdSSS with existing scales (Functional Oral Intake Scale (FOIS), Functional Intake LEVEL Scale (FILS), and the unified MSA rating scale (UMSARS)) using Spearman's rank correlation coefficients. Sixty-three patients were evaluated by videofluorographic (VF) swallowing examination. To evaluate concurrent validity, Spearman's rank correlation coefficients were calculated between the NdSSS and VF swallowing assessments. Additionally, scale responsiveness was determined using the standardized response mean (SRM) in 23 patients who could be followed up to assess their long-term course. Study 2: Cross-sectional survey of swallowing function and history of pneumonia. Data regarding history of pneumonia, UMSARS, NdSSS, age, sex, MSA subtype, and disease duration were retrospectively obtained from the medical records of 113 patients with MSA. Differences in these parameters and NdSSS stage between those with and without a history of pneumonia were examined using the Mann-Whitney test or chi-squared test. Furthermore, clinical factors related to a history of pneumonia were examined by binomial logistic regression analysis.

**Results:**

The NdSSS showed satisfactory reliability, concurrent validity, and responsiveness. A history of pneumonia was related to the severity of MSA, age, MSA subtype, and NdSSS stage. Binomial logistic regression analysis showed that NdSSS stage (odds ratio (OR), 0.490; 95% confidence interval (CI), 0.301–0.797, *p* = 0.001) and MSA subtype (OR, 4.031; 95% CI, 1.225–13.269, *p* = 0.021) were significantly associated with a history of pneumonia.

**Conclusions:**

In patients with MSA, the NdSSS has sufficient reliability, concurrent validity, and responsiveness for assessing dysphagia. Patients with a history of pneumonia have more severe dysphagia. We found that the pneumonia risk was related to NdSSS stage and MSA-p (predominantly parkinsonism). Meticulous care to prevent aspiration is needed from early stages of the disease.

## Introduction

Multiple system atrophy (MSA) is a sporadic and rapidly progressive neurodegenerative disorder that presents with autonomic failure in combination with parkinsonism or cerebellar ataxia (1). The main features of MSA include parkinsonism, cerebellar signs, autonomic symptoms, and pyramidal signs (1–3). In addition, dysarthria, stridor (1–5), urinary dysfunction, and dysphagia can also occur (1, 2, 4, 5). Among the symptoms of MSA, dysphagia is perhaps the most clinically significant (2, 4, 6), because it can result in silent aspiration and, consequently, pneumonia (4, 6), which has been reported as the leading cause of death in MSA (2, 5, 7–9). Thus, evaluation of swallowing function is crucial in patients with MSA.

Dysphagia is one of the most serious problems in patients with progressive neuromuscular diseases. However, there are no standardized evaluation procedures for early screening for dysphagia in these patients (10). A systematic review of the screening and assessment of dysphagia in neuromuscular disease patients stated that most studies found that videofluorographic (VF) evaluation of swallowing was an effective and reliable test for assessing dysphagia (10). However, in patients with progressive neuromuscular disease, it is necessary to evaluate swallowing function in daily life with minimal burden on the patient. Recently, the Neuromuscular disease Swallowing Status Scale (NdSSS) (11) was developed to assess specific swallowing disorders in patients with progressive neuromuscular diseases (11) and is currently widely used (10, 12). The NdSSS has been shown to have satisfactory reliability, validity, and responsiveness, and can be used in both myogenic and neurogenic disorders, whether the speed of disease progression is rapid or slow. However, its use has so far only been reported for Duchenne muscular dystrophy (DMD) (11), Becker muscular dystrophy (13), and amyotrophic lateral sclerosis (ALS) (11), but not for MSA. Yet, it is possible that the NdSSS might be useful for evaluating swallowing function in MSA patients without burdening them.

There have been few detailed reports regarding pneumonia in patients with MSA (4, 6, 7, 14, 15). These previous reports noted that evaluation and adequate treatment of dysphagia might prevent or delay complications, such as aspiration pneumonia, and increase survival time (4). Thus, despite considerable concern about aspiration pneumonia in patients with MSA, there are few reports on the direct relationship between dysphagia and pneumonia. Additionally, none of these previous reports used a binomial logistic regression analysis to examine the relationship between dysphagia and pneumonia.

The aim of this study was (1) to investigate whether the NdSSS is a convenient tool for assessing swallowing function in patients with MSA, with evaluation of its interrater and intrarater reliabilities, concurrent validity, and responsiveness, and (2) to identify the factors related to a history of pneumonia in patients with MSA. To achieve these goals, we divided the study into two parts: Study 1 was performed to evaluate the interrater and intrarater reliability, concurrent validity, and responsiveness of the NdSSS for assessing dysphagia in MSA patients, and Study 2 was performed to examine whether dysphagia is involved in the development of pneumonia.

## Materials and Methods

### Participants

The participants were 129 inpatients with MSA. The patients were recruited from April 2012 to August 2017 at National Hospital Organization Higashi Saitama National Hospital. All patients who were admitted for evaluation of general condition and advanced disability and referred to the Department of Rehabilitation Medicine were enrolled. Cases in which VF evaluation was not performed during hospitalization were excluded. The study was approved by the Ethics Committee of National Hospital Organization Higashi Saitama National Hospital (#13–29) and was performed in accordance with the Declaration of Helsinki. The Ethics Committee approved a method of obtaining consent in accordance with the relevant guidelines, without requiring written consent, by making the study outline available to the public and guaranteeing participants the opportunity to withdraw from the study.

### Study 1: Reliability, Concurrent Validity, and Responsiveness of the NdSSS in Patients With MSA

#### Reliability

The sample for the interrater and intrarater reliability studies included 81 patients with MSA, of whom 63 had MSA-c (predominantly cerebellar involvement) and 18 had MSA-p (predominantly parkinsonism). For the interrater reliability study, two physiatrists trained in the use of the NdSSS independently assessed the patients. For the intrarater reliability study, one physiatrist assessed the patients using the NdSSS twice on two separate days.

### Concurrent Validity

Spearman's rank correlation coefficient was used to assess concurrent validity of the NdSSS, by correlating its stages with those of other existing swallowing function clinical scales (swallowing item of the unified MSA rating scale (UMSARS Sw) (2), Functional Oral Intake Scale (FOIS) (16), and the Functional Intake LEVEL Scale (FILS) (17)) measured at the same time. The FOIS (16) is a clinical scale for stroke with dysphagia that has been shown to have adequate reliability, validity, and sensitivity (16). The FILS (17) is a practical tool to assess the severity of dysphagia in various diseases associated with dysphagia. It has fair reliability and validity (17). The unified MSA rating scale (UMSARS) (2) is used to measure the level of physical functioning in activities of daily living (ADL) in patients with MSA. To evaluate concurrent validity, Spearman's rank correlation coefficient for the severity of MSA on the Global Disability Scale (GDS) in UMSARS (2) and the swallowing function clinical scales (NdSSS, FOIS, FILS, and UMSARS Sw) was calculated in patients with MSA.

Sixty-three [54 MSA-c/ 9 MSA-p, mean age ± SD 66.1 ±5.5 years, disease duration mean ± SD 5.3 ± 3.0 y, GDS median (range) 3 (1–5)] of 81 patients with MSA in Study 1 were evaluated by VF swallowing examination. The VF examination was performed between 1 and 2 weeks after admission, using 5 mL of a semi-liquid with a viscosity of 150–300 mPa∙s. To evaluate concurrent validity, Spearman's rank correlation coefficients were calculated between the NdSSS and VF swallowing assessments using the Penetration–Aspiration scale (P–A scale) (18) and videofluorographic dysphagia scale (VDS) (19). The P–A scale is used to describe penetration and aspiration events (18), and the VDS is one of the videofluorographic scales for patients with dysphagia due to a variety of etiologies (19, 20). The VDS and PAS results were reviewed and interpreted by a single specialist certified by the Japanese Association of Rehabilitation Medicine, who had more than 10 years of experience with swallowing disorders in patients with neurodegenerative diseases at the time of the study.

### Responsiveness

The responsiveness of each of the NdSSS, FILS, FOIS, and UMSARS Sw was determined by calculating effect sizes from the first visit to the follow-up visit. Several methods have been proposed to quantify the responsiveness of outcome measures. The standardized response mean (SRM), which is the mean change in the score for a scale divided by the standard deviation of the change in scores, allows us to make statistically meaningful comparisons between the instruments. A higher SRM indicates a greater effect or clinically important change, with SRMs of 0.2, 0.5, and 0.8 and above representing small, moderate, and large clinical changes, respectively (21, 22).

In 23 patients with MSA [disease duration (mean ± SD) 4.4 ± 3.2 y, UMSARS part IV median (range) 2 (1–5)], the NdSSS, FILS, FOIS, and UMSARS Sw were assessed twice in about 14 months, and the SRM was calculated to quantify each instrument's responsiveness to short-term changes. The average follow-up period was 416.7 (SD 181.5, range 144–959) days.

#### Study 2: Cross-Sectional Survey of Swallowing Function and History of Pneumonia

**Figure 1:**
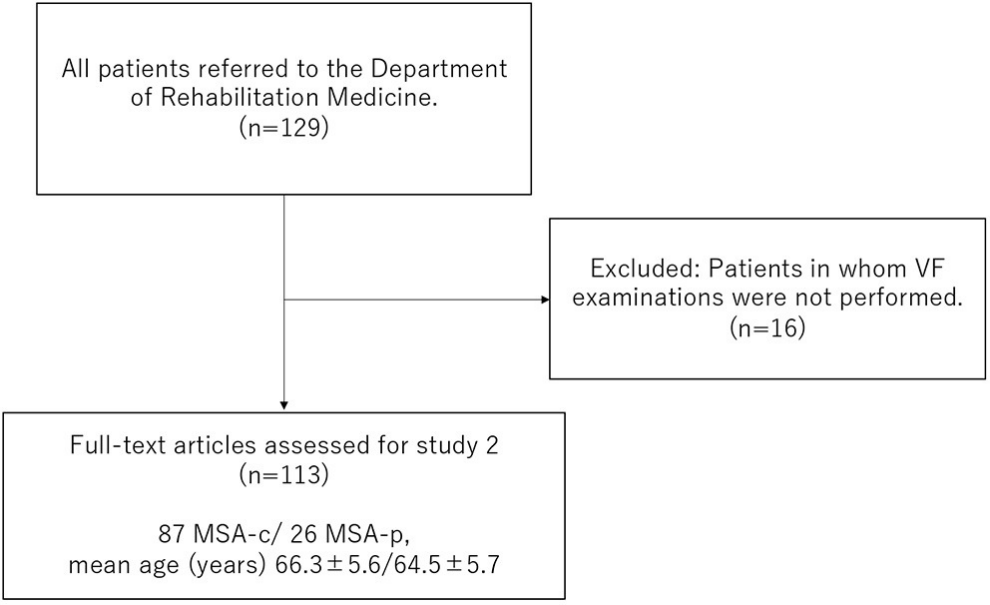
Flow diagram of study 2.

Information on 113 patients with MSA [87 MSA-c/ 26 MSA-p, mean age ± SD 65.9 ±5.6 years, disease duration (mean ± SD) 5.0 ± 2.9 y, GDS median (range) 3 (1–5)] related to their age, sex, disease duration, GDS in UMSARS, and history of pneumonia within 1 year of VF examination, was extracted retrospectively from their medical records (Figure 1). The presence of pneumonia was diagnosed by the neurologist based on clinical symptoms and imaging examinations as the presence of acute infiltrate on a chest radiograph or chest CT (23–25). At the same time, dysphagia was assessed using the NdSSS, and its relationship to a history of pneumonia was investigated.

### Statistical Analyses

#### Study 1: Reliability, Concurrent Validity, and Responsiveness of the NdSSS in Patients With MSA

Interrater and intrarater reliabilities were examined for each item using weighted kappa statistics. To assess the concurrent validity of the NdSSS, the correlations between the NdSSS and FOIS, FILS, and UMSARS Sw in patients with MSA were assessed using Spearman's rank correlation coefficients. In addition, the correlations between the GDS in UMSARS and NdSSS, FILS, FOIS, and UMSARS Sw in patients with MSA were also assessed using Spearman's rank correlation coefficients. The correlations between the NdSSS and the P–A scale and VDS were also assessed using Spearman's rank correlation coefficients. Responsiveness was examined using SRMs.

#### Study 2: Cross-Sectional Survey of Swallowing Function and History of Pneumonia

The differences in clinical information (age, disease duration, and GDS) and the NdSSS stage between those with and without a history of pneumonia were examined by the Mann-Whitney test. However, the chi-squared test was performed to examine the relationship between sex and MSA subtypes with a history of pneumonia. The items with significant differences in the above results were assessed for multicollinearity and used as explanatory factors, and binomial logistic regression analysis of the history of pneumonia was performed.

All analyses were performed with a significance level set at < 0.05. Data analyses were performed using JMP, version 13 (SAS Institute Inc., Cary, NC, USA).

## Results

### Study 1: Reliability, Concurrent Validity, and Responsiveness of the NdSSS in Patients With MSA

Patient demographics and their relevant clinical features are summarized in Table 1.

**Table 1:** Demographics of the study 1 sample.

		**MSA-c**	**MSA-p**
Number		63	18
Mean age ± SD (y)		66.5 ± 5.2	67.2 ± 7.9
Sex			
Male		28	6
Female		35	12
Disease duration (y)		6.6 ± 5.0 (1–22)	6.4 ± 2.7 (4–16)
UMSARS Part IV		
Median (min-max)		4 (1–5)	4.5 (1–5)

*MSA-c, Multiple system atrophy (predominantly cerebellar involvement); MSA-p, Multiple system atrophy (predominantly parkinsonism); SD, standard deviation; UMSARS, unified MSA rating scale*.

#### Reliability

The weighted kappa for the NdSSS was 0.99 for both interrater and intrarater reliability.

#### Concurrent Validity

Table 2 shows Spearman's rank correlation coefficients between the NdSSS and FOIS, FILS, and UMSARS Sw. The NdSSS showed strong correlations with all three previous scales. Table 3 shows Spearman's rank correlation coefficients between the Global Disability Scale in UMSARS and NdSSS, FOIS, FILS, and UMSARS Sw. Significant correlations were observed between the swallowing scales and the severity of physical function scales. Table 4 shows the results of VF swallowing assessments and their correlations with the NdSSS. Significant correlations were seen between the NdSSS and both the VDS and P–A scale in patients with MSA.

**Table 2:** Spearman's rank correlation coefficients between the Neuromuscular disease Swallowing Status Scale and other dysphagia clinical scales.

	**Spearman's rank correlation coefficient (** ***ρ*** **)**			
	**MSA-c (*N* = 63)**	***p*-value**	**MSA-p (*N* = 18)**	***p*-value**
FOIS	0.88	<0.0001	0.96	<0.0001
FILS	0.96	<0.0001	1	<0.0001
UMSARS Sw	−0.82	<0.0001	−0.89	<0.0001

*MSA-c, Multiple system atrophy (predominantly cerebellar involvement); MSA-p, Multiple system atrophy (predominantly parkinsonism); FOIS, Functional Oral Intake Scale; FILS, Food Intake LEVEL Scale; UMSARS Sw, unified MSA rating scale Swallow*.

**Table 3:** Spearman's rank correlation coefficients between the Global Disability Scale and swallowing function clinical scales according to disease subtype.

	**Spearman's rank correlation coefficient (ρ)**			
	**MSA-c (N = 63)**	***p*-value**	**MSA-p (N=18)**	***p*-value**
NdSSS	−0.8	*p* <0.001	−0.94	*p* <0.001
FOIS	−0.73	*p* <0.001	−0.92	*p* <0.001
FILS	−0.81	*p* <0.001	−0.94	*p* <0.001
UMSARS Sw	0.84	*p* <0.001	0.88	*p* <0.001

*MSA-c, Multiple system atrophy (predominantly cerebellar involvement); MSA-p, Multiple system atrophy (predominantly parkinsonism); NdSSS, Neuromuscular Disease Swallowing Status Scale; FOIS, Functional Oral Intake Scale; FILS, Food Intake LEVEL Scale; UMSARS Sw, unified MSA rating scale Swallow*.

**Table 4:** Spearman's rank correlation coefficients between the Neuromuscular Disease Swallowing Status Scale and videofluorographic swallowing assessments.

	**Spearman's rank correlation coefficient (ρ)**	***p*-value**
P–A scale	−0.46	0.0002
VDS	−0.58	<0.0001

*P–A scale, Penetration-Aspiration Scale; VDS, videofluorographic dysphagia scale*.

#### Responsiveness

SRMs for the NdSSS, FILS, FOIS, and UMSARS Sw were 0.65, 0.53, 0.39, and 0.50, respectively. SRM was higher for NdSSS than for the other scales.

### Study 2: Cross-Sectional Survey of Swallowing Function and History of Pneumonia

Table 5 compares clinical information and NdSSS stage between patients with and without a history of pneumonia. A history of pneumonia was related to MSA severity and NdSSS stage regardless of disease duration. The chi-squared test also showed that MSA subtype was related to a history of pneumonia. On VF examination, the total VDS score was 12.5 (0–55.5) for the group with no history of pneumonia and 39 (0–73.5) for the group with a history of pneumonia. The oral phase scores for those with no pneumonia and with pneumonia were 4.5 (0–30) and 9 (0–25.5) respectively, and the pharyngeal phase scores for those with no pneumonia and with pneumonia were 6.5 (0–41) and 26 (0–51.5), respectively. A history of pneumonia was related to aspiration and oral and pharyngeal phase dysfunction. The results indicated differences in clinical factors and swallowing function between those with and without a history of pneumonia. On univariate analysis, a history of pneumonia was associated with not only swallowing status, but also dysphagia on VF examination (Table 6).

**Table 5:** Difference in clinical information and NdSSS stage by history of pneumonia in patients with multiple system atrophy.

		**No pneumonia**	**With pneumonia**	***p*-value**
Number (%)		90 (79.6)	23 (20.4)	
Sex (M/F)		47/43	12/11	0.997
Subtypes (c/p)		73/17	14/9	0.049
Age (y)		65.3 ± 5.3	68.2 ± 6.3	0.038
Disease duration (y)		4.8 ± 2.9 (1–15)	5.8 ± 3.0 (1–16)	0.054
GDS in UMSARS				
Median (min-max)		3 (1–5)	4 (1–5)	0.002
NdSSS				
Median (min-max)		7 (3–8)	6 (1–7)	<0.001

*VF, videofluorographic; GDS, Global Disability Scale; UMSARS, unified MSA rating scale; NdSSS, Neuromuscular disease Swallowing Status Scale*.

**Table 6:** Difference in swallowing function by history of pneumonia in patients with multiple system atrophy.

		**No pneumonia**	**With pneumonia**	***p*-value**
Number (%)		90 (79.6)	23 (20.4)	
Sex (M/F)		47/43	12/11	0.997
Subtype (c/p)		73/17	14/9	0.049
Age (y)		65.3 ± 5.3	68.2 ± 6.3	0.038
Disease duration (y)		4.8 ± 2.9 (1–15)	5.8 ± 3.0 (1–16)	0.054
GDS in UMSARS			
Median (min-max)		3 (1–5)	4 (1–5)	0.002
NdSSS				
Median (min-max)		7 (3–8)	6 (1–7)	<0.001
P–A scale				
Median (min-max)		1 (1–8)	1 (1–8)	0.02
VDS				
Median (min-max)				
Total score		12.5 (0–55.5)	39 (0–73.5)	<0.001
Oral phase		4.5 (0–30)	9 (0–25.5)	<0.001
Pharyngeal	phase	6.5 (0–41)	26 (0–51.5)	<0.001

*VF, videofluorographic; GDS, Global Disability Scale; UMSARS, unified MSA rating scale; NdSSS, Neuromuscular disease Swallowing Status Scale; P–A scale, Penetration-Aspiration Scale; VDS, videofluorographic dysphagia scale*.

Table 7 shows the results of binomial logistic regression analysis in relation to a history of pneumonia. The dependent variable was a history of pneumonia, and the explanatory variables were age, MSA subtype, the GDS in UMSARS, and NdSSS stage, based on the results shown in Table 5. Analysis showed that NdSSS stage and MSA subtype were significantly associated with a history of pneumonia. The odds ratio showed that a lower NdSSS, i.e., more advanced dysphagia, indicated a greater likelihood of development of pneumonia in both MSA-p and MSA-c patients.

**Table 7:** Binomial logistic regression analysis of factors related to a history of pneumonia in patients with multiple system atrophy.

**Factors**	**OR**	**95%CI**	***p*-value**
Subtype (ratio of MSA-p to MSA-c)	4.031	1.225–13.269	0.021
Age (per 1 year old)	1.102	0.993–1.223	0.058
GDS in UMSARS (per 1 point)	1.336	0.833–2.143	0.226
NdSSS (per 1 level)	0.490	0.301–0.797	0.001

*GDS, Global Disability Scale; UMSARS, unified MSA rating scale; OR, odds ratio; CI, confidence interval*.

Table 8 shows the differences in clinical factors and swallowing function between the MSA subtypes. For clinical factors and VF swallowing examination, the differences between MSA-c and MSA-p were examined using the Mann-Whitney test. A history of pneumonia and distribution of MSA subtypes was evaluated by the chi-squared test. Evaluation indicated no difference between the MSA subtypes in clinical factors and penetration and aspiration events. However, swallowing function on the VDS was significantly worse for MSA-p than for MSA-c in terms of all three: the oral phase score [MSA-p/MSA-c: 6.8 (0–23)/4.5 (0–33)], pharyngeal phase score [MSA-p/MSA-c: 22.3 (0–45.5)/7.5 (0–51.5)], and total score [MSA-p/MSA-c: 31.8 (0–55)/12.5 (0–73.5)]. Additionally, the number of patients with a history of pneumonia was significantly lower in MSA-c (14 of 87, 16%) than in MSA-p (9 of 26, 35%; chi-squared *p* = 0.049).

**Table 8:** Differences between MSA subtypes in terms of clinical evaluations and videofluorographic swallowing assessments.

		**MSA-c**	**MSA-p**	***p*-value**
Number		87	26	
Sex				
Male		42	17	
Female		45	9	
Mean age ± SD (y)		66.3 ± 5.6	64.5 ± 5.7	0.129
Disease duration (y)		5.1 ± 3.1 (1–16)	4.7 ± 2.2 (1–9)	0.923
UMSARS Part IV			
Median (min-max)		3 (1–5)	3 (1–5)	0.517
NdSSS				
Median (min-max)		7 (1–8)	7 (1–8)	0.143
P–A scale				
Median (min-max)		1 (1-8)	1 (1–8)	0.231
VDS				
Median (min-max)				
Total score		12.5 (0-73.5)	31.8 (0–55)	0.011
Oral phase		4.5 (0–33)	6.8 (0–23)	0.041
Pharyngeal phase		7.5 (0–51.5)	22.3 (0–45.5)	0.007
History of pneumonia		14 (16.1%)	9 (34.6%)	0.049

*MSA, multiple system atrophy; VF, videofluorographic; SD, standard deviation; UMSARS, unified MSA rating scale; NdSSS, Neuromuscular disease Swallowing Status Scale; P–A scale, Penetration-Aspiration Scale; VDS, videofluorographic dysphagia scale*.

## Discussion

This study identified factors associated with aspiration pneumonia in patients with MSA. This is the first report to examine factors associated with a history of pneumonia using binomial logistic regression analysis, and included the largest number of pneumonia cases when compared with previous studies. This study showed that pneumonia in patients with MSA is associated with NdSSS stage and MSA-p. In other words, pneumonia in patients with MSA was predominantly associated with dysphagia and with disease type. Furthermore, the reliability, validity, and responsiveness of the NdSSS were shown, suggesting the utility of NdSSS in assessing dysphagia in patients with MSA.

The NdSSS was developed as a versatile tool for the assessment of swallowing disorders in neuromuscular diseases that cause progressive dysphagia; in this study, we tested its reliability, validity, and responsiveness to determine its suitability for clinical use in the context of neurodegenerative diseases, such as MSA. The results of the present study demonstrated that NdSSS, a simple swallowing disorders assessment for progressive neuromuscular disease, has satisfactory reliability, validity, and responsiveness in patients with MSA. VF examination and UMSARS-Sw are generally used to evaluate swallowing function in patients with MSA, although there are also reports of use of the Dysphagia and Outcome Severity Scale (26–28) and the American Speech-Language-Hearing Association's National Outcome Measurement System (20, 29). The present study also found that the NdSSS had superior reliability, validity, and responsiveness to the other existing scales. Since the NdSSS has greater stratification than the other scales, it enables more detailed evaluation of swallowing disorders in MSA patients. The advantages of the NdSSS are that it can be assessed in a short time without the need for a special device for evaluation (11), and that it is can easily capture changes in swallowing function over a long period of time wing to its level of detail. The NdSSS stage correlated well with VF parameters and was found to be a major risk factor for pneumonia. Thus, while VF examination is useful for detailed functional evaluation in the management of dysphagia in patients with MSA, the NdSSS is useful as a scale that allows for the convenient evaluation of dysphagia at the bedside or at home without special equipment.

Dysphagia is feared as a cause of aspiration pneumonia in patients with progressive neuromuscular diseases (10). However, there have been few detailed reports addressing pneumonia in these patients, other than those evaluating the association between aspiration pneumonia and swallowing function by univariate analysis (6). So far, there have been no reports showing that pneumonia is associated with aspiration in patients with neuromuscular diseases, including neurodegenerative diseases such as MSA. Therefore, this report demonstrating the relationship between pneumonia and dysphagia using binomial logistic regression analysis is valuable as it provides the basis for the establishment of evaluation methods for dysphagia, which might contribute to preventing aspiration in patients with MSA. Additionally, the finding that swallowing dysfunction is a direct cause of pneumonia in patients with MSA is consistent with previous clinical reports (4, 6, 7, 14, 15), and shows that it is necessary to adopt methods that will allow ease in following the evolution of swallowing function during the patients' clinical course, in order to prevent the development of aspiration pneumonia. We believe that the NdSSS is a useful tool for easily identifying dysphagia.

With respect to MSA subtypes shown to be a risk factor for pneumonia in patients with MSA, parkinsonism is more likely to be associated with pneumonia than ataxia. Patients with Parkinson's disease and parkinsonism are known to have a high risk of pneumonia (15, 30, 31). In parkinsonism, respiratory function, and especially cough function, is considered to be related to pneumonia risk. Coughing is an airway defense mechanism designed to clear the airway and prevent aspiration (32, 33). In addition, the increased risk of pneumonia might also be due to chest wall rigidity and the diminished cough reflex in patients with Parkinson's disease (15, 30, 34). On the other hand, it has been reported that simple ataxia alone does not increase the risk of pneumonia (35). We believe this is consistent with the present report. However, further studies are needed to measure and compare cough parameters by MSA subtype.

As in a previous report showing that the most common dysphagia symptom was aspiration in both MSA subtypes (29), the oral and pharyngeal phases were impaired in both subtypes in the present VDS results. However, swallowing function with VDS was significantly worse for MSA-p than for MSA-c in both the oral and pharyngeal phases, and the incidence of pneumonia was also higher in MSA-p patients. A previous study evaluating VDS showed that the amount of vallecular residue was greater in patients with MSA-p than in those with MSA-c (29), which has been described as signifying reduced tongue base retraction and pharyngeal contraction in MSA-p than in MSA-c (19). Previous reports supporting our results showed that MSA-p patients had reduced or absent duration of inhibition of cricopharyngeal muscle activity (36), and hypertonic swallow-related muscle tone due to parkinsonism (37). Abnormal upper esophageal sphincter resting pressure may also be relevant (38). Certainly, MSA-p might cause aspiration pneumonia due to impaired swallowing function for the above reasons. Thus, MSA-p is more likely to cause severe dysphagia and is more likely to cause aspiration pneumonia than MSA-c, and meticulous care to prevent aspiration, as well as provision of pulmonary rehabilitation and exercise programs are necessary from an early stage of the disease.

There are several limitations to the present study. First, the study was conducted at only one institution; thus, the results must be generalized with caution. Second, the number of MSA-p subjects was smaller than that of MSA-c patients. Unlike in Europe and the United States (1), in Japan it is reported that MSA-c is about twice as frequent as MSA-p (39). In the present study, the proportion of MSA-c was higher (77%), and it was about 3.3 times more common than MSA-p (23%). However, although the number of patients was small, we believe that the overall picture of MSA was captured because disease severity varied from mild to severe in both subtypes. Third, in the present study, MSA subtypes were divided according to the initial symptoms (14), and no consideration was given to the overlapping of advanced stage parkinsonism and cerebellar ataxia symptoms with disease progression. In fact, some of the patients recruited in this study were in an advanced stage, and it was not possible to compare cases purely representing either parkinsonism or cerebellar ataxia cases. Despite these limitations, the findings of the present study provide important information for the management of patients with MSA. We believe the results of this research will help inform the prevention or management of the development of pneumonia in patients with MSA.

## Conclusion

The NdSSS has satisfactory reliability, validity, and responsiveness in patients with MSA, and is a useful method for evaluating dysphagia in patients with MSA. Binomial logistic regression analysis in this study showed that NdSSS stage and MSA-p are risk factors for pneumonia in patients with MSA. Thus, meticulous care to prevent aspiration is needed from an early disease stage.

## Data Availability Statement

The raw data supporting the conclusions of this article will be made available by the authors, without undue reservation.

## Ethics Statement

The studies involving human participants were reviewed and approved by Ethics Committee of National Hospital Organization Higashi Saitama National Hospital (#13-29). Written informed consent for participation was not required for this study in accordance with the national legislation and the institutional requirements.

## Author Contributions

AW and MK were mainly responsible for conception and design of the study, data analyses and interpretation, and drafting the manuscript. KK, NH, and FL contributed to data acquisition, analysis, and interpretation in this study. YY, TO, and TT contributed to the conception and design of the study. All authors contributed to the article and approved the submitted version.

## Conflict of Interest

The authors declare that the research was conducted in the absence of any commercial or financial relationships that could be construed as a potential conflict of interest.

## Publisher's Note

All claims expressed in this article are solely those of the authors and do not necessarily represent those of their affiliated organizations, or those of the publisher, the editors and the reviewers. Any product that may be evaluated in this article, or claim that may be made by its manufacturer, is not guaranteed or endorsed by the publisher.
